# The Anticancer Activity of Cannabinol (CBN) and Cannabigerol (CBG) on Acute Myeloid Leukemia Cells

**DOI:** 10.3390/molecules29245970

**Published:** 2024-12-18

**Authors:** Ahmad Kadriya, Sarah Forbes-Robertson, Mizied Falah

**Affiliations:** 1 Medical Research Institute, The Holy Family Hospital Nazareth, Nazareth 16100, Israel; ahmadkadriya@gmail.com; 2 Azrieli Faculty of Medicine, Bar-Ilan University, Safed 1311502, Israel; 3 Health & Life Sciences Department, Coventry University, Coventry CV1 5FB, UK; ac2690@coventry.ac.uk

**Keywords:** leukemia cells, anticancer activity, cannabinoids, cannabinol (CBN), cannabigerol (CBG), enocannabinoid system (ECS)

## Abstract

Several cannabis plant-derived compounds, especially cannabinoids, exhibit therapeutic potential in numerous diseases and conditions. In particular, THC and CBD impart palliative, antiemetic, as well as anticancer effects. The antitumor effects include inhibition of cancerous cell growth and metastasis and induction of cell death, all mediated by cannabinoid interaction with the endocannabinoid system (ECS). However, the exact molecular mechanisms are still poorly understood. In addition, their effects on leukemia have scarcely been investigated. The current work aimed to assess the antileukemic effects of CBN and CBG on an acute monocytic leukemia cell line, the THP-1. THP-1 cell viability, morphology and cell cycle analyses were performed to determine potential cytotoxic, antiproliferative, and apoptotic effects of CBN and CBG. Western blotting was carried out to measure the expression of the proapoptotic p53. Both CBN and CBG inhibited cell growth and induced THP-1 cell apoptosis and cell cycle arrest in a dose- and time-dependent manner. CBN and CBG illustrated different dosage effects on THP-1 cells in the MTT assay (CBN > 40 μΜ, CBG > 1 μM) and flow cytometry (CBN > 5 μM, CBG > 40 μM), highlighting the cannabinoids’ antileukemic activity. Our study hints at a direct correlation between p53 expression and CBG or CBN doses exceeding 50 μM, suggesting potential activation of p53-associated signaling pathways underlying these effects. Taken together, CBG and CBN exhibited suppressive, cell death-inducing effects on leukemia cells. However, further in-depth research will be needed to explore the molecular mechanisms driving the anticancer effects of CBN and CBG in the leukemia setting.

## 1. Introduction

Acute myeloid leukemia (AML) is a deadly disease generally occurring in later adulthood (17 per 100,000 persons above the age of 65 years) but also diagnosed among youth (1.8 per 100,000 persons below the age of 15 years) [1,2]. Up to date analysis shows a growing global burden of AML, particularly in higher Socio-Demographic Index regions, highlighting its impact across all age groups [3]. AML is considered fatal if left untreated, and the median survival is estimated to be between 11 and 20 weeks after diagnosis [1,4]. As defined, AML consists of eight subtypes (from M0–M7) based on cell morphological and cytochemical features. Its pathogenesis compromises the abnormal proliferation of immature immune cells derived from the myeloid cells originating from hematopoietic stem cells in the bone marrow, characterized by somatic alterations and chromosomal rearrangements, such as t(8:21) or t(15:17) and other epigenetic mutations that carry significant implications regarding prognosis, like DNMT3A and IDH-1 or IDH-2 genes [5]. Due to such genetic alterations in the hematopoietic stem cells in the AML disease, the bone marrow and the peripheral blood frequently show more than 20% myeloblast immature cells with expressed hematopoiesis-associated antigens like CD34, CD38, and CD33 [6].

Phytocannabinoids, the bioactive molecules present in cannabis, have been under intensive research for the last decade, mainly due to their neuropharmacological and psychotropic effects in many animal species as well as in the human biological system [7]. Strong evidence of the anti-anxiety, anti-inflammatory, anti-glaucoma, anti-epileptic, antiemetic effects, and analgesic properties of cannabinoids has been documented [8,9,10,11]. However, clinical research has not fully elucidated the mechanisms of their action and still debates their uses under certain pathological conditions, mainly due to the psychotropic side effects of some cannabinoids [10,12]. More than 120 cannabinoid molecules have been extracted from cannabis, among which 9-tetrahydrocannabinol (THC) and cannabidiol (CBD) are the most abundant molecules and have been found to elicit different pharmacological and physiological actions [10,11,13]. These molecules could control cell fate through both endocannabinoid system (ECS) and non-ECS receptors [14]. These molecules are lipophilic ligands that inherently interact with the ECS through G protein-coupled cannabinoid receptors (CB1 and CB2), which are widely expressed in human cells [15,16], and they could cross the blood–brain barrier (BBB) and target brain cancerous cells [17]. Upon binding to their receptors, exogenous or endogenous cannabinoids can trigger signaling under normal and pathological conditions [16,18]. Exogenous plant-derived cannabinoids share properties with endogenous ligands (endocannabinoids) produced in the human body, such as arachidonoylethanolamide (anandamide) and 2-arachidonoyl glycerol, which function as neuromodulators, immunomodulators, and retrograde synaptic messengers [19,20,21]. Their impact on various chronic diseases, especially cancer, has been demonstrated in a broad spectrum of cancer cells, and they were categorized as antimetastatic, antiproliferative, antiangiogenic, as well as apoptotic cannabinoids [22]. THC exerts a cytotoxic effect on brain cancer cell lines as well as on healthy brain cell lines; however, it only induces apoptosis in cancer cells via the sphingomyelin cycle (elevation of ceramide levels), with no effect on healthy cells [23]. THC was also found to induce apoptosis in rat cortical neurons by phosphorylating the p53 apoptotic cascade via the CB1 receptor [24]. Similarly, CBD was found to inhibit breast cancer cell growth and metastases both in vitro and in vivo by enhancing reactive oxygen species (ROS) levels [25]. It was suggested to cause ceramide accumulation in breast cancer cells through the interaction with the CB2 receptor, which subsequently induces apoptosis [25]. Moreover, CBD, in lung cancer cells, was shown to induce apoptosis by activating the caspase-3 cascade [26].

In light of the aforementioned studies, cannabinol (CBN) and cannabigerol (CBG) have recently gained great attention for their therapeutic potential. The two molecules are classified as non-psychotropic plant-derived cannabinoids and were both shown to interact with the CB1/CB2 receptors [27]. CBN, the oxidized form of THC, is a CB2 receptor agonist, thereby modulating the downstream signaling of cyclic adenosine monophosphate (cAMP) and also activates the TPRM8 channels by antagonizing the receptor. CBG, on the other hand, is called the “stem cell” of most phytocannabinoids, as it converts to other cannabinoids during plant growth and is a nonpsychoactive cannabis-derived cannabinoid. Although CBG is categorized as a weak agonist of the CB receptors due to its low affinity, it has been shown to be a potent TRPM8 antagonist [13]. A recent study suggested that CBG, only in certain types of cells, can partially agonize the CB2 receptor, consequently leading to ERK phosphorylation and β-arrestin recruitment while demonstrating limited noncompetitive engagement of the CB1 receptor [28]. CBG was observed to inhibit colon carcinogenesis; higher potency of CBG was observed in cells expressing non-endocannabinoid receptors [29]. More specifically, CBG fully antagonized the transient receptor potential member 8 (TRPM8), which regulates cancer cell proliferation/apoptosis by inducing ROS overproduction, without interacting with endocannabinoid receptors CB1 or CB2. This indicates that some cannabinoid groups can have pro-apoptotic effects on certain cells and tissues regardless of the levels of CB receptor expression. Several investigations have also shown the effects of cannabinoids when paired with antileukemic drugs. The combination of CBG, CBD, or THC with conventional antileukemic drugs reduced drug cytotoxicity and even brought a significant improvement in the overall antileukemic effect on the human cancer cell lines, such as CEM (ALL) and HL60 (acute promyelocytic leukemia, M3). This suggests that mixing cannabinoids can elevate their anticancer effects, proposing a possible entourage effect between different cannabinoids [30]. Similar outcomes of cannabinoid pairing were shown in a prostate cancer model (PC3 prostate cancer cell line), where cannabis extract containing CBN, CBD, and THC significantly reduced the viability and growth of these cells [31]. Thus, the anticancer activities of cannabinoids are remarkably complex, stimulating cells differently depending on the disease setting, as their downstream signaling mechanisms are diverse and are strongly connected to the cannabinoid affinity to the receptor [32].

One of the notable and novel discoveries that opened a new research area regarding the antileukemic potential of cannabinoids was the cloning of the G protein-coupled receptor CX5 (CB2) by Munro and his group [33]. They reported that, unlike CB1, the CB2 receptor is predominantly localized on immune cells and plays a more specific role in regulating intracellular immune mechanisms. Subsequent experiments have further characterized the role of the CB2 receptor in cellular signaling pathways, particularly its regulatory effects on various immune cell types.

In the leukemia setting, cannabinoids were observed to block cancer cells at an early stage of their cell maturation and to inhibit growth and proliferation, primarily due to their interaction with the CB2 cannabinoid receptor.

A study investigating the effects of natural and synthetic exogenous cannabinoids on malignant lymphoblastic disease both in vivo and in vitro demonstrated that THC and JWH-015 induced apoptosis in tumor cells in a dose-dependent, CB2 receptor-dependent manner. Treatment with 5 mg/kg THC significantly increased the survival of C57BL/6 mice. Moreover, peripheral blood-isolated acute lymphoblastic leukemia (ALL) cells exhibited marked apoptosis within 2 h of THC exposure at various concentrations. Dronabinol (THC extracted from the marijuana plant) is a commercial drug used to treat the side effects caused by cancer chemotherapy, such as nausea and vomiting. It demonstrated antileukemic activity in acute lymphoblastic and myeloid leukemia models [34]. Dronabinol abrogated the proliferation of Jurkat (T-cell leukemia) and MOLM13 (AML cell line) cells and induced apoptosis via the cannabinoid receptors CB1 and CB2, demonstrating that the response to certain cannabinoids may involve both G-coupled receptors. However, a previous study measured low CB1 receptor expression in some leukemic cell lines, like Jurkat, thereby dismissing the possible involvement of CB1 in the induction of apoptosis [35]. Powles and colleagues [36] demonstrated THC concentration-dependent stimulation of cell killing in CEM (acute lymphoblastic), HEL-92 (M6 acute myeloid leukemia), HL60, and MOLT-4 (acute lymphoblastic) leukemic cell lines via the mitogen-activated protein kinase (MAPK) pathway, which is believed to inactivate ERK2, subsequently triggering apoptosis. The effect was still observed in the presence of CB receptor antagonists, suggesting a CB-independent cell death mechanism. Similarly, CBD, the non-psychoactive cannabinoid, demonstrated the potency to activate the caspase-8, -9, -3 and to increase the ROS production in Jurkat and Molt-4 leukemia cell lines, suggesting the possibility of extrinsic apoptotic pathways [37].

As part of our ongoing efforts to investigate the anticancer effects of cannabinoids on malignant immune cell lines and to develop new therapeutic approaches, the current work aims to study the antileukemic effects of CBN and CBG on the THP-1 cell line, an acute monocytic leukemia (subtype M5). Specifically, their effects on cell viability, cell cycle, proliferation, and cell morphology were evaluated, as was the potential relationship between their effects on cell viability and p53-driven apoptosis.

## 2. Results

### 2.1. The Effect of CBN and CBG on THP-1 Cell Viability

**Figure 1:**
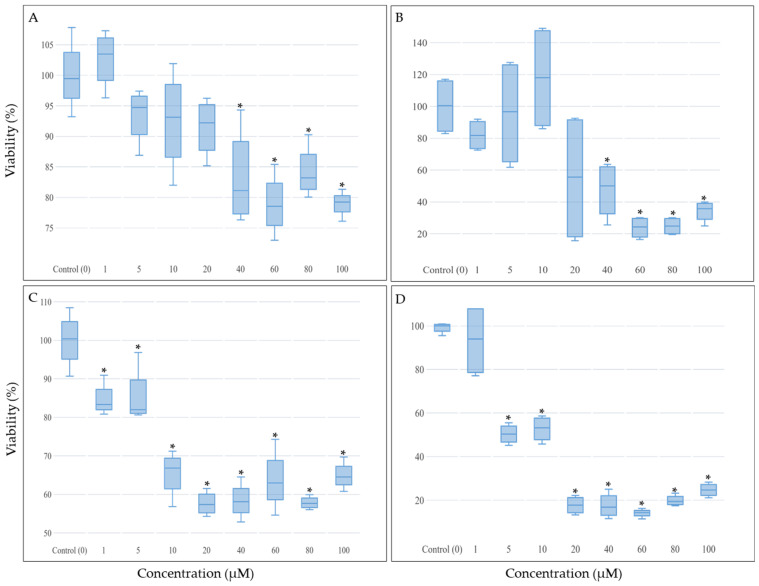
The effect of CBN and CBG on THP-1 cell viability. (**A**,**C**) indicate the effect of various concentrations of CBN and CBG, respectively, on THP-1 cells after 24-h incubation. (**B**,**D**) indicate the effect of the increasing concentrations of CBN and CBG, respectively, on THP-1 cells after 48-h incubation. The star (*) indicates a *p*-value of *p* < 0.05, which was considered statistically significant between the control (culture medium without treatment) and the increasing concentrations using CBG or CBN.

To examine the cytotoxic effects of CBN and CBG on THP-1 viability, cells were cultured with increasing concentrations of each cannabinoid and then subjected to the MTT assay. Both CBN and CBG reduced THP-1 cell viability in a dose- and time-dependent manner within the range of 0–100 µM (Figure 1), with CBG having a greater effect (*p*-values < 0.05). After a 48-h exposure to either of the cannabinoids at doses exceeding 20 µM, less than 50% of the cells remained viable (Figure 1B,D).

The data shown were normal and had a homogeneity of variances. The star (*) indicates a *p*-value of *p* < 0.05, which was considered statistically significant between the control and the increasing concentrations. While CBN decreased cell viability starting from 40 μM, CBG showed a significant decrease at all concentrations compared to the control. Hence, CBG reduced cell viability more effectively than CBN in a dose-dependent manner after 24-h incubation. After 48 h, CBN and CBG showed a more significant reduction in viability (less than 50% live cells).

### 2.2. The Effect of CBN and CBG on THP-1 Cell Cycle and Proliferation

**Figure 2:**
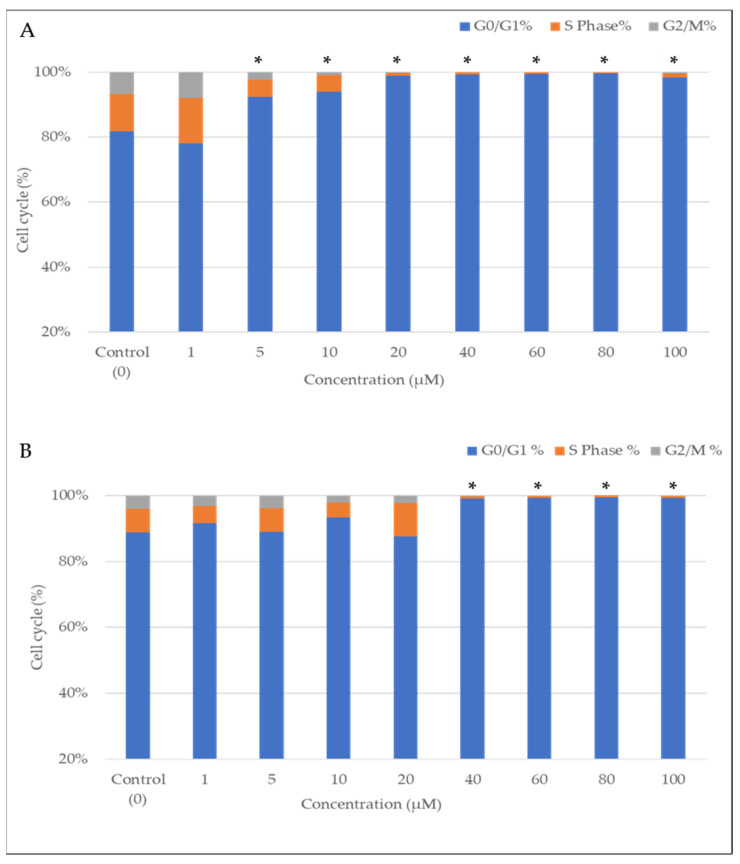
The effects of CBN and CBG on the cell cycle of THP-1 cells. Chi-squared analysis was performed to assess the significance of differences between the control and the increasing concentrations of (**A**) CBN- or (**B**) CBG-treated cells. The star (*) represents a *p*-value < 0.05 compared to the control (cell with culture medium only). The data shown here is a typical experiment repeated three times (*n* = 3).

To determine whether CBN or CBG can inhibit cell proliferation via cell cycle arrest, cell cycle analysis based on the quantitation of DNA content was performed using flow cytometry and cell staining with propidium iodide (PI). Treatment with either CBN or CBG resulted in a dose-dependent accumulation of cells in the G0/G1 phase (Figure 2), with CBN exerting a stronger cell cycle arrest effect (Figure 2A). More specifically, the percentage of cells in the G2/M and S phases was significantly reduced after treating cells with ≥5 μM CBN or with ≥40 μM CBG (Figure 2A,B).

Based on the fluorescence from the propidium iodine stain in each phase (G0/G1, S, and G2/M phases), the flow cytometer analysis showed a difference in the cell cycle distribution compared with the control in both CBN and CBG. CBN led to a significant decrease in the S and G2/M phase and arrested cells in the G0/G1 phase following treatment with 5 μM concentration and above, while CBG induced arrest starting at a 40 μM concentration.

**Figure 3:**
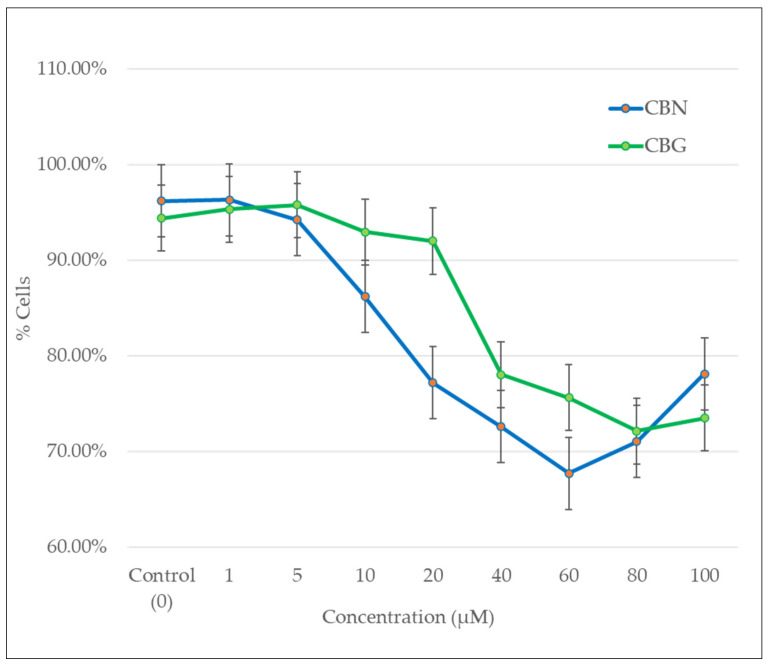
The effects of CBN and CBG on the cell percentage of THP-1 cell population in SSC and FSC scatter analysis. The presented data are the average of triplicate samples in three different experiments.

In the forward and side scatter analysis (Figure 3), the number of cell populations gradually decreased with increasing concentrations of CBN or CBG. The linear-scale and forward-scatter analysis showed that both cannabinoids reduced cell population compared to the control, with CBN already showing an effect at lower concentrations.

Flow cytometry analysis of the percentage of cells in the S/G2–M phase indicated a gradual decrease in the number of stained cells (as manifested by DNA fluorescence from propidium iodine) with increasing concentrations of CBG or CBN (0–100 μM). CBN showed a more significant effect in reducing cells in the S or G2/M phase and in arresting cells in the G0/G1 phase compared to CBG.

### 2.3. Structural Alterations in THP-1 Cells Induced by CBN and CBG Treatment

**Figure 4:**
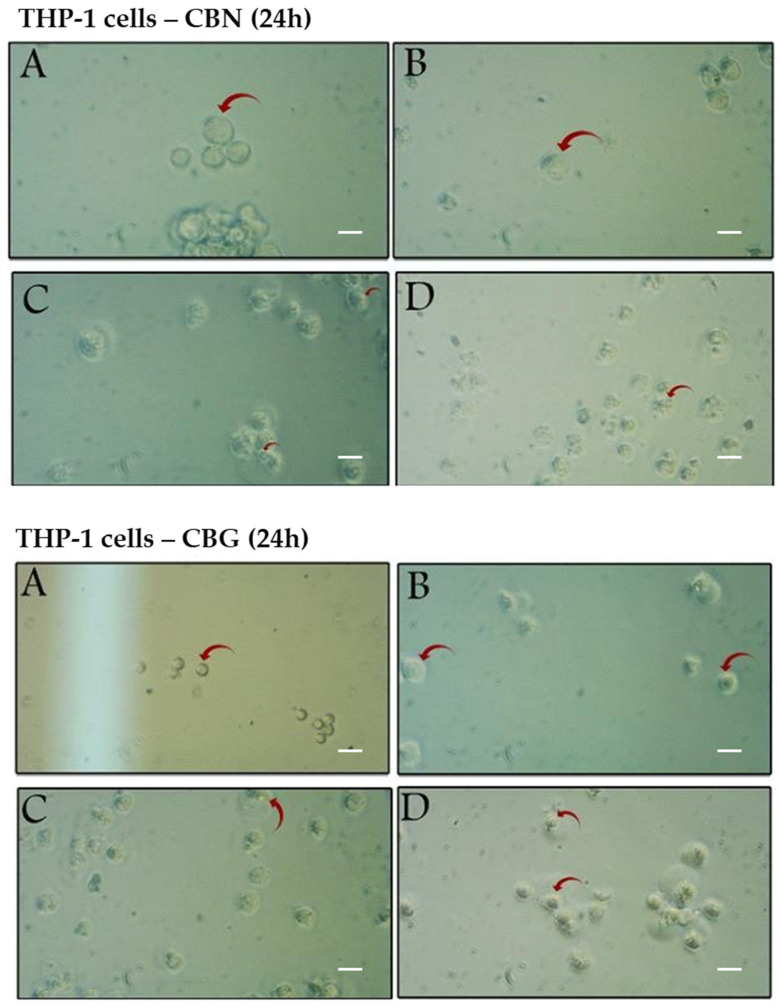
Effect of CBG or CBN treatment on THP-1 cell morphological characteristics. THP-1 cells were treated with CBN (**upper panel**) or CBG (**lower panel**) for 24 h at concentrations of 0 µM (**A**), 1 µM (**B**), 50 µM (**C**), and 100 µM (**D**). The red arrows mark changes in morphological characteristics in THP-1 cells in comparison with the control 0 µM (**A**). Representative fields were imaged at 200× magnification. Scale bar = 25 µm.

**Figure 5:**
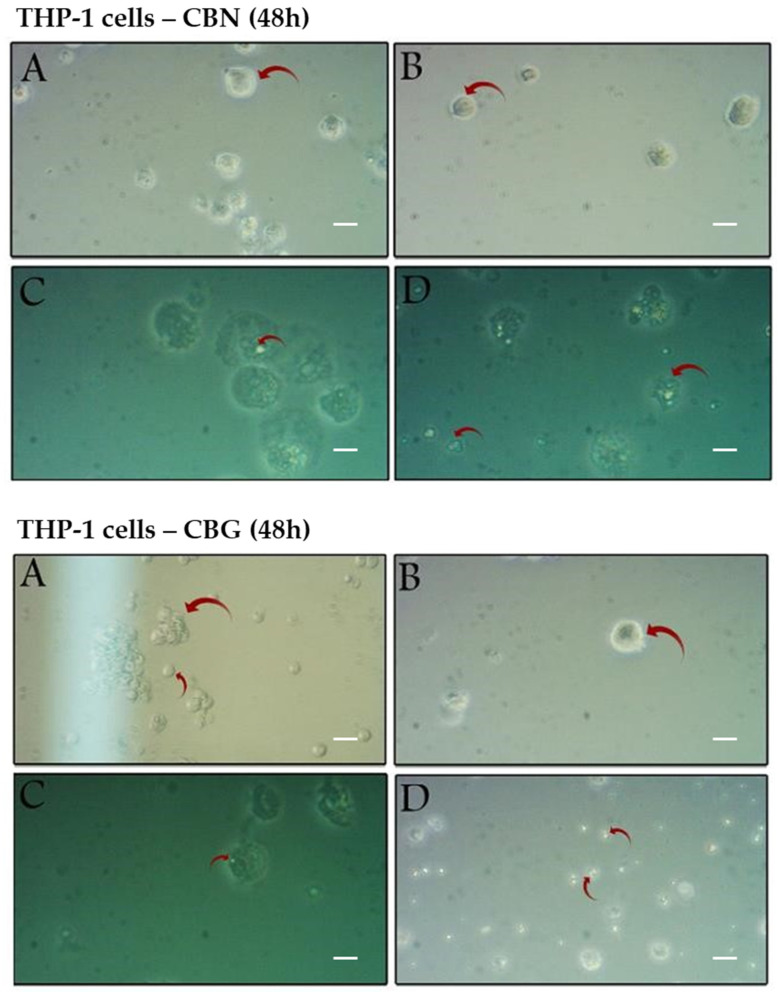
Effect of CBG or CBN treatment on THP-1 cell morphological characteristics. THP-1 cells were treated with CBN (**upper panel**) or CBG (**lower panel**) for 48 h at concentrations of 0 µM (**A**), 1 µM (**B**), 50 µM (**C**), and 100 µM (**D**). The red arrows mark changes in morphological characteristics in THP-1 cells in comparison with the control 0 µM (**A**). Representative fields were imaged at 200× magnification. Scale bar = 25 µm.

Morphological changes in THP-1 cells following 24- or 48-h exposure to CBN or CBG were analyzed using light microscopy. Notable dose- and time-dependent reductions in growth and proliferation of cancer cells were demonstrated (Figure 4 and Figure 5). After 24 h of incubation with CBN or CBG (Figure 4), the control cells and those treated with 1 μM cannabinoids had a smooth membrane with a circular nucleus, which resembled monoblastic cells (Figure 4A,B). In contrast, cells treated with higher concentrations of cannabinoids (Figure 4C,D) were deformed and exhibited early signs of chromatin and cytoplasmic condensation, likely with a shift toward apoptosis. Cytoplasmic vacuolation, which usually accompanies cell death, was also observed (Figure 4C).

As seen, cells treated with a low concentration of CBN or CBG for 24 h showed no significant morphological changes, while treatment with higher and increasing concentrations of CBN or CBG led to cytoplasmic vacuolization, swelling, and a significant reduction in cell cytoplasmic density. However, following 48-h exposure to CBN or CBG, cells treated with the lowest tested dose (1 μM) still did not demonstrate any observable cytopathic effects (Figure 5B), compared with the untreated cells (Figure 5A), which were able to proceed with cell division and become more confluent. At 50 μM of either cannabinoid, far fewer live cells were present compared with the 24-h treatment (Figure 5C), and cytoplasmic vacuolization and condensation, along with distinguished membrane ruptures, were observed. At the highest dose tested (Figure 5D), the number of apoptotic cells increased, as demonstrated by nuclear fragmentation and abundant cell debris.

Taken all together, the apoptotic events induced by cannabinoids are strongly dose- and time-dependent, clearly demonstrating morphological features in THP-1 cells, including clear vacuolization in the cytoplasm, breakdown of cellular structures, and notable cellular fragmentation.

### 2.4. The Effect of CBN or CBG on p53 Expression in Treated THP-1 Cells

**Figure 6:**
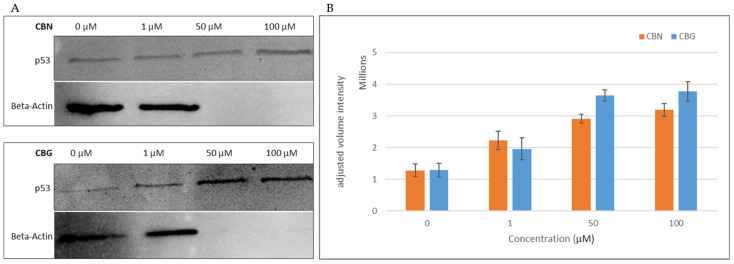
Western blot analysis of p53 expression in THP-1 leukemia cells following cannabinoid treatment. Expression of p53 in cells treated with CBN (**upper panel**) and CBG (**lower panel**) for 24 h with 0, 1, 50, and 100 μM is shown. Beta-actin bands were used as a loading control, as indicated in (**A**). Relative to the untreated control (0 μM), both CBG and CBN treatments induced an increase in p53 protein levels at 1, 50, and 100 μM, with maximal intensity observed at 50 and 100 μM. Notably, beta-actin expression was absent at 50 and 100 μM. The band’s volume intensity was quantified via the optical density of each band, as indicated in (**B**).

p53 serves as a critical regulator of cell survival. To examine the association between p53 modulation in response to CBG or CBN treatment in THP-1 leukemia cells, the cells were treated with increasing concentrations (0, 1, 50, and 100 μM) and incubated for 24 h (Figure 6). The findings reveal a dose-dependent correlation between cannabinoid concentration and p53 expression, as indicated by an increase in band intensity relative to the control. Furthermore, at concentrations above 50 μM, beta-actin expression was undetectable, suggesting cytoskeletal impairment within the cells due to the cannabinoid’s treatment.

## 3. Discussion

Cannabinoids and their derivatives have been the subject of scientific and clinical investigations and debates for decades, mostly due to their diverse effects on disease symptoms and complications as well as their excitant action on the nervous system [10,11,21,38]. As documented, accumulated evidence has demonstrated their antitumor activities in numerous cancer models [9,22,39]. However, their therapeutic potential for some malignancies, particularly leukemias, remains relatively unknown. The efficacy of conventional treatments for acute leukemias is largely unsatisfactory for most entities; therefore, this study was undertaken to investigate the antileukemic properties of plant-derived cannabinoids CBN and CBG in the leukemia THP-1 cell line. This study demonstrated the anticancer, antileukemic potential of plant-derived compounds that have not yet been thoroughly examined.

Neither the therapeutic nor the toxic dosage for CBN and CBG has been established in vitro or in vivo [40]; hence, the current work focused on a concentration range that is clinically achievable and well-tolerated based on previously conducted leukemia research with THC and CBD [30,34,37]. The presented in vitro data suggest that CBN and CBG can induce apoptosis in THP-1 cells and inhibit their growth in a dose- and time-dependent manner (Figure 1). After the 24-h incubation (Figure 1A,C), CBG proved more effective in reducing the viability of THP-1 cells than CBN. However, after a 48-h incubation, the impact of the cannabinoids on cell viability was more significant, indicating that the activity of both cannabinoids is time-dependent (Figure 1B,D). The notable differences observed between the two incubation periods were supported by the microscopical analysis of cell morphology. After 24-h incubation, the cytotoxic effects of CBN and CBG were less observable (Figure 4B–D), with cells treated with 50 μM or 100 μM showing early apoptotic features but without complete cell death. After a 48-h incubation, later features of cell death were noted (Figure 5B–D). More cells were alive at 24 h as compared with 48 h (Figure 4), which can hint that cells have not necessarily completed the programmed cell death cycle.

The flow cytometric analysis demonstrated that CBN and CBG also had antiproliferative effects. After 24-h incubation with 5 μM CBN or 40 μM of CBG, the percentage of cells in the S and G2/M phases was significantly reduced (<1%) compared with the control, indicating that CBN is more efficient in restraining cell growth (Figure 2). It is important to note that the significant arrest of cells in G0/G1 at higher concentrations of CBN or CBG may be associated with apoptosis, as suggested by microscopic examinations and forward scatter analysis. After a 24-h incubation with the cannabinoids, changes in cell shape, cell shrinkage, and a decrease in living cells (Figure 4C,D) were observed, while the percentage of cells gradually declined with increasing concentrations of the cannabinoids (Figure 3). These findings by both cannabinoid effects suggest the involvement of multiple mechanisms and pathways.

The microscopic analysis of cannabinoids-treated THP-1 cells identified morphological alterations, which differed by CBN/CBG concentration and incubation duration. It was previously shown that some cannabinoid groups at a particular concentration range have a distinctive mode of action in hematological malignancies [41]. Investigating immunophenotypic markers (CD markers antibodies) or immune staining to detect blast maturation induced by cannabinoids can provide additional evidence of their activity. Such analyses will likely support the results obtained from the MTT assay and flow cytometry analysis.

One of the aims of this study was to investigate the effect of CBG and CBN on p53 expression in THP-1 cells. To our knowledge, currently, the effect of cannabinoids on p53 apoptotic signaling in leukemic cells hasn’t been completely elucidated, especially in the THP-1 cell line. The western blotting analysis demonstrated a pronounced increase in expression of p53 at concentrations of 50 and 100 μM compared with the control (Figure 6). At concentrations below 50 μM, no bands were detected of p53 in the western blot. The p53 status in the THP-1 cell line was reported to contain a partial deletion, 26 bases from the 1st letter of codon 174 [42], and the sequence length was observed to be shorter than the wildtype. It is well established that p53 activity and function in tumors are often suppressed in various cancer types [43]. However, in some types of cancers, like hematological malignancies, certain pathways become dysfunctional, triggering p53, and the truncated versions of the p53 protein can still perform aspects of their tumor-suppressor role. A similar finding to that of our study, where p53 expression increased in leukemic cells (Jurkat clone E6-1 cells carry a homozygous mutation in the p53 gene at codon 143), was observed in an investigation on the effect of the synthetic cannabinoid CP55940 [44]. The study showed that although Jurkat cells contain the mutated form, p53 was still regulated by the cell at different concentrations. Moreover, an attempt to inhibit the activity of the cannabinoid used resulted in no change in p53 expression, highlighting the cannabinoid’s mode of action on CB1 and CB2 receptors. Notably, beta-actin, the housekeeping gene employed in this assay, exhibited undetectable expression levels at higher concentrations of both cannabinoids. To further explore this observation, cells were treated with concentrations below 50 μM. At these lower concentrations, beta-actin expression was detectable; however, at 40 μM, the corresponding band was faintly visible. This outcome is further corroborated by post-treatment microscopic images (Figure 4 and Figure 5), which display pronounced cellular morphological alterations indicative of apoptotic processes. Beta-actin is a critical component of the cellular cytoskeleton, playing a key role in migratory behavior and cytoskeletal dynamics. Additionally, the actin family is essential in initiating apoptosis due to its involvement in maintaining cellular integrity and driving morphological changes [45]. However, comprehensive data on the direct effects of cannabinoids on the actin family remain limited. Nonetheless, several anticancer agents and compounds have been documented to target the actin cytoskeleton and corresponding pathways [46]. The Western blot results suggest a potential mechanism of action for CBN and CBG involving both the p53 pathway and the actin family.

The mechanism of action of cannabinoids is generally described as involving their interaction with endocannabinoid receptors (CB1/CB2 receptor), which changes the receptor state and induces numerous signaling pathways inside the cell [47]. In several studies on hematological malignancies, the CB2 receptor has been the primary target due to its selective localization on immune cells. However, the underlying molecular anticancer mechanism of most cannabinoids remains poorly understood [48,49]. THC showed dose-dependent induction of apoptosis in leukemia cells (Jurkat and Sup-T1 cell lines) through the binding of CB2 receptors [35]. The anticancer efficiency of THC was also observed to increase in correlation with the incubation period, suggesting that cannabinoids are also time-dependent in their effects. Dronabinol was also shown to confer antileukemic activity against lymphatic and myeloid leukemia cell lines [34] and to induce apoptosis in Jurkat and MOLM13 cell lines in a dose-dependent manner, with a concentration of 75 μM completely killing cells while 15 μM (THC IC-50 concentration) only inhibited cell growth. A mix of cannabis extracts was observed to reduce the viability of the leukemia cell lines HL-60 and CEM at a concentration range of 1–50 μM after 48 h [30]. The study proposed that treating cancer cells with combined cannabinoids yields a more substantial anticancer effect with lower doses, as compared to a single compound at higher concentrations. The potency embodied by responses elicited by cannabinoids depends on the CB receptor expression level, and this can explain the different doses required for different cannabinoids to trigger specific effects [21,50]. CBN and CBG are both considered partial agonist ligands of the CB2 receptor but with different binding affinities [13,28]. Since the CB2 receptor is predominantly expressed in immune cells, it can be speculated that the associated anticancer effects induced by CBN and CBG on THP-1 cells are CB2 receptor-dependent. Cannabinoids that engage with the CB2 receptor are believed to trigger several signaling pathways that mostly involve MAPK, ion channels, adenylyl cyclase and beta-arrestin [51]. The signaling pathway by which cannabinoids induce p53 activation, as documented in the literature, is through the MAPK (Mitogen-Activated Protein Kinase) pathway, which includes p38, JNK, and ERK [27]. These kinases are capable of phosphorylating p53, thereby regulating its activity. Building on this understanding, the MAPK pathway plays a critical role in regulating p53 activity, particularly through phosphorylation events that enhance its stability and transcriptional activity, thereby promoting apoptosis in cancer cells [52]. This suggests that the engagement of CB2 receptors by CBN and CBG may activate MAPK signaling, which in turn could mediate the observed upregulation of p53 in THP-1 cells, further supporting their targeted anticancer effects. Only a small pool of leukemia studies has reported changes in leukemia cell machinery due to exposure to cannabinoids [49]. It has been demonstrated that THC upregulated the DUSP gene that encodes MKP3 in three different human acute leukemia cell lines (i.e., CEM, HL60, and MOLT-4) [36]. MKP3 is known to regulate members of the MAPK/ERK, stress-activated proteins that control cell survival. These signals were suggested to be triggered via the CB2 receptor. Exposure of Jurkat leukemia cells to THC led to apoptosis through the intrinsic and extrinsic apoptotic pathways [53]. Specifically, THC activated caspase-8 and caspase-10 as part of the extrinsic pathway and induced the cleavage of caspase-2 and caspase-9 as part of the intrinsic (mitochondrial) pathway. THC and other synthetic cannabinoid CB2 receptor agonists were shown to trigger apoptotic and antiproliferative mechanisms in various cancer models, including leukemic and nonleukemic malignancies. CBN and CBG are less potent compounds compared to THC and other synthetic agonists of the CB2 receptor [13], yet it is speculated that their antiproliferation and apoptotic effects are mediated via similar cellular mechanisms. It is important to note that the results of the viability assay (24-h incubation) and cell cycle analysis (Figure 1 and Figure 2) reflect the variability in potency of the CB receptor between CBN and CBG in THP-1 cells. Arguably, both techniques can be considered as measurements of proliferation and apoptosis, based on the fact that in some cancer cells, the induction of apoptosis is dependent on the cell cycle pathways [54]. However, CBN, unlike CBG, was previously suggested to have a unique mode of action in leukemia cell lines [41]. At low concentrations (30 μM), CBN induced differentiation, but not apoptosis, in a human myeloblastic leukemia cell line (ML-2 cell line); the CBN-treated cells expressed maturation markers and phenotypes in a dose- and time-dependent manner. This finding may explain the inhibition of THP-1 proliferation by lower doses of CBN as compared to CBG (Figure 2 and Figure 3).

Cannabinoids, unlike conventional cancer drugs, were shown in numerous cancer models to elicit apoptosis and inhibit cell division via different signaling pathways [14]. In addition, cannabinoids are being used in modern medicine to treat side effects associated with chemotherapy administration, such as chemotherapy-induced peripheral neuropathy and chemotherapy-induced nausea and vomiting [55]. Based on previous findings concerning the activity of cannabinoids in leukemia models, the working hypothesis in the current study was that the effects of CBN and CBG on THP-1 are mediated by their interaction with the CB2 receptor [35,36]. Yet, CBN and CBG can also induce CB2 receptor-independent anticancer effects. To determine the role of the cannabinoid receptors in the response to CBN or CBG, a CB2 receptor antagonist, such as SR144528 (a synthetic cannabinoid), can be added together with CBN or CBG [36]. Alternatively, CB2 receptor expression can be silenced using shRNAs or siRNA. This approach can explain the behavior of CBN and CBG in the presence of additional cannabinoids, knowing that the endocannabinoid system consists of endogenous cannabinoids such as N-arachidonoylethanolamine (AEA) and 2-arachidonoylglycerol (2AG) [20]. The available in vivo and clinical data on cannabinoids as a treatment for cancer, especially leukemia, is still limited. Currently, the only published human trial testing any cannabinoid in an oncological setting was a small clinical pilot study in which THC was intracranially injected in patients with recurrent glioblastoma [56], and a phase 1b clinical trial of Nabiximols, a cannabinoid spray containing THC and CBD, was conducted in patients with recurrent GBM [57]. A study reported on a 14-year-old patient diagnosed with aggressive Philadelphia-positive ALL who took a cannabinoid extract (hemp oil) showed significant reductions in leukemic blast cell counts measured at different periods [58]. Specifically, after 15 days of cannabinoid treatment, the blast count dropped from 250,000 to 61,000, and after 39 days, the blast count dropped dramatically to 2000. Although observational, a dose-dependent response was achieved with minimal side effects.

Overall, the results suggest that the non-psychotropic cannabinoids CBN and CBG hold promise as agents for regulating cancer cell survival and growth in a leukemia context. The widespread recreational use of cannabis underscores its perceived safety profile, particularly regarding cellular toxicity and damage. Its long history of use, supported by growing scientific evidence, suggests that cannabinoids primarily exhibit antinociceptive effects through their interaction with the central nervous system and possess immunomodulatory properties with minimal side effects [59,60]. Our findings pave the way for novel investigations into the unique mechanisms of cannabinoids across diverse cancer models, as their effects may vary depending on specific cellular abnormalities. Although preliminary, this study supports the potential for further in vitro and in vivo research to elucidate the anticancer activity of these cannabinoids on leukemic cells, potentially contributing to the development of a new class of anticancer agents.

## 4. Materials and Methods

### 4.1. Cell Culture

The human leukemia cell line THP-1, an acute monocytic leukemia M5, was kindly donated by Dr. Bernard Burke (Coventry University, UK). Cells were cultured and maintained in RPMI 1640 culture medium, supplemented with 1 mmol/L L-glutamine, 10% serum (*v*/*v*) fetal bovine serum (FBS), and 1% (*w*/*v*) penicillin/streptomycin (Sigma Aldrich, Gillingham, UK). The cell line was incubated in a humidified atmosphere with 5% CO_2_ and 95% O_2_ at 37 °C. The density was maintained between 2 × 10^5^–1 × 10^6^ cells/mL, and the medium renewal was done every two days to avoid cell differentiation, according to the ATCC protocol. The viability of cells was assessed using a trypan blue stain of 0.4% (Thermofisher, Horsham, UK).

### 4.2. Cannabinoids and Cell Treatment

CBN was purchased from Sigma (Sigma Aldrich, Gillingham, UK) with a stock concentration of 1 mg/mL (% *w*/*v*) in methanol, CBG crystal powder (0.5 g, 97% purity) was purchased from Pharma Hemp (PharmaHemp, Barcelona, Spain). Cannabinoid concentrations during all experiments were carried out in molar concentrations. The CBN stock concentration was converted to molarity (3.22 mM) and was serially diluted. A total amount of 33 mg of CBG was dissolved in 1 mL of 100% dimethyl sulfoxide (DMSO), obtaining a final concentration of 100 mM. The study investigates the effect of CBN and CBG in a concentration range of 1 to 100 μM. To exclude toxic effects on cells, the concentrations of methanol and DMSO were maintained below 1% throughout all experiments. The concentration range of CBG and CBN (1–100 µM) employed in this study was selected based on prior in vitro research, aligning with established experimental frameworks to facilitate comparative analysis and validation of results [61]. Furthermore, studies have consistently demonstrated that cannabinoids exhibit binding affinities and physiological effects within the micromolar range, particularly in interactions with CB1, CB2, and related receptor systems [28,62].

### 4.3. Cell Viability Assay (MTT)

To investigate the cytotoxic effects of CBN and CBG on viable THP-1 cells, the MTT assay was adopted according to previously described protocols with minor adjustments [30,63]. Initially, cells were seeded in 96-well plates overnight at 37 °C and a 5% CO_2_ incubator to allow cells to settle at the bottom of the wells. Cells were seeded at a density of 3 × 10^4^ cells/well and treated with cannabinoids for 24 and 48 h, respectively, with increasing concentrations (0–100 μM CBG/CBN). The experiment control (0) consisted of cells in a culture medium without treatment. The concentrations were made in a final volume of 200 µL. After treatment, 20 µL of 5 mg/mL 3-4,5-dimethythiazol 2-yl-2,5-diphenyl-2H-tetrazolium bromide (MTT) (Sigma Aldrich, Gillingham, UK) reagent was added and left for 2 h incubation to allow the formazan product to form in live cells. Plates were then centrifuged at 800 RPM for 5 min, and 200 µL of 100% DMSO was added to solubilize the formazan crystals product from the cells and left for 10 min incaution at room temperature. The dissolved formazan product was measured spectrophotometrically using a plate reader (LT-4500 SPECTRO, software T-COM V7.0, Spectra Services, Ontario, NY, USA), and the readings obtained were calculated according to the MTT method [64] using the formula: viability percentage (%) = (OD of treated cells/OD of untreated cells (control)) × 100.

### 4.4. Cell Cycle Analysis by Flow Cytometry

The cell cycle profile of THP-1 cells was determined using Propidium iodide (PI) stain (Sigma Aldrich, UK), a fluorescence dye that stains DNA stoichiometrically, allowing the differentiation of cells in G0/G1, S phase, and G2/M. Flow cytometric analysis was done based on a previously described method, with a few adjustments [65,66]. THP-1 cells were seeded at a density of 20,000 cells/well in 24-well plates and grown overnight. Cells were then treated with CBN and CBG for 24 h at a concentration range of 0–100 μM. Untreated/unstained cells served as a negative control for cannabinoids and the PI stain. Cells were transferred to FACS tubes, gently mixed with a micropipette to reduce cell clumping, and then centrifuged and washed twice with PBS. After washing, the cell pellet was vortexed, fixed with cold 70% ethanol and incubated on ice for 30 min. Cells were then centrifuged at 4000 rpm for 2 min, and ethanol was removed. The cell pellet was resuspended in a staining buffer containing 0.05% Triton x-100, 20 µg/mL PI solution, and 10 µg/mL RNase (Sigma Aldrich, Gillingham, UK) and incubated for 1 h in the dark at room temperature. The percentage of cell cycle phases, namely, G0/G1, S and G2/M, was determined for 10,000 events at low speed using BD Accuri™ C6 Plus, software, FACS v3.1 (BD Biosciences, Erembodegem, Belgium) in the FL3 channel (488-nm Blue/Green laser line). Cellular debris and clumped cell regions were gated out, and stained cells were viewed on a linear scale and forward scatter.

### 4.5. Microscopic Analysis

To confirm the cytotoxic effects of CBN and CBG, intracellular and extracellular morphological changes were examined by light microscopy. Cells at a density of 8 × 10^4^ cells/well were seeded in 24-well plates and exposed to CBN or CBG at 1, 5, 50, and 100 μM concentrations. The concentration of each cannabinoid was made in a final volume of 500 µL. The plates were incubated for 24 and 48 h and then examined under the inverted light microscope at 40×/20×.

### 4.6. Western Blot Analysis for p53 Protein Expression

To assess the effect of CBN and CBG on p53 expression, 4 × 10^5^ cells were seeded in a 24-well plate in duplicates. Cells were left for overnight incubation, followed by cannabinoid exposure at concentrations of 1, 50, and 100 μM in a final volume of 250 μL. Cannabinoid absence in wells served as a negative control. All plates were then incubated for 24 h in an incubator set to 37 °C and 5% CO_2_. Protein extraction, separation, and antibody probing were performed according to a previously described method [67].

#### 4.6.1. Protein Extraction and Quantification

Treated and untreated cells were centrifuged at 4000 rpm for 5 min. The culture medium was discarded, and the cell pellet was washed with 500 µL cold PBS. A total of 100 µL of RIPA lysing buffer (150 mM NaCl, 0.1% Triton X100, 0.5% sodium deoxycholate, 0.1% SDS, 50 mM Tris-HCl, pH 8.0, protease inhibitors) (Sigma Aldrich, Gillingham, UK) was then added to each cell pellet and incubated on ice for 30 min. The lysate was centrifuged at 4000 rpm for 10 min, followed by transferring the supernatant to a new tube and discarding the pellet. Protein concentration in the supernatant samples was quantified using a spectrophotometer Nanodrop ND-1000, software Nanodrop-1000 V3.8.1 (Thermofisher, Horsham, UK) and blanked with RIPA buffer. Following protein concentration determination for each cell lysate, samples were further diluted to a working concentration of 100 μg/mL in 100 μL RIPA buffer. This adjustment was made to achieve a final concentration of 50 μg/mL protein by mixing with 2X Laemmli sample buffer in a 1:1 ratio (%*v*/*v*) (protein to 2X Laemmli buffer) (Bio-Rad, Watford, UK).

#### 4.6.2. Gel Electrophoresis

Cell lysate samples containing 50 μg of protein in 2X Laemmli sample buffer were heated at 95 °C for 3 min to denature the proteins before loading on two gels. A total of 5 μL of protein ladder (10–250 kD, Bio-Rad, Watford, UK) was loaded into the wells of a sodium dodecyl sulfate-polyacrylamide gel electrophoresis (SDS-PAGE, any KD Mini-PROTEAN TGX Precast Protein, Bio-Rad, UK). In the remaining wells, 19 μL of cell lysate buffer was loaded. The gels were run at 130 V for 1 h.

#### 4.6.3. Protein Transfer to a PVDF Membrane

Proteins from the gels were transferred to a Trans-Blot Turbo PVDF membrane (Bio-Rad, Watford, UK) using the Trans-Blot Turbo Transfer System at 12 A for 10 min, according to the manufacturer’s protocol (Bio-Rad, Watford, UK).

#### 4.6.4. Primary and Secondary Antibody Probing

The primary monoclonal antibodies, anti-p53 (clone, DO-7, mouse igG2b, Thermofisher, Horsham, UK), and anti-beta-actin (clone BA3R, anti-mouse igG2b, Thermofisher, Horsham, UK) as a loading control, and the secondary antibody (horseradish peroxidase-conjugated anti-mouse immunoglobulin G1n, Thermofisher, Horsham, UK) were diluted according to the manufacturer’s protocol (Thermofisher, Horsham, UK). The membranes were blocked for 1 h at room temperature with 5% milk in TBST (Tris-buffered saline and Tween 0.25%, 15 mL final volume) in a Falcon tube. First, the membrane was incubated with anti-p53 (1:200 dilution in 20 mL TBST), and after identifying the expression, the membrane was incubated with anti-beta-actin (1:200 dilution in 20 mL TBST) for 24 h at 4 °C. After incubation, the membranes were washed three times for 5 min with TBST and then reincubated with the secondary antibody (1:1000 dilution in 2 mL TBST) for 1 h at room temperature. The three-step wash was also performed after the secondary antibody incubation. Immunoreactive bands were treated with 2 mL of high-sensitivity Chemiluminescence substrate (Thermofisher, Horsham, UK) and detected by Biorad, Image-Lab software v6.1, according to the manufacturer’s protocol (Bio-Rad, Watford, UK).

### 4.7. Statistical Analysis

All statistical analyses were performed using SPSS (SPSS, software v25) or Microsoft Excel (office 365). The data from the MTT assay were analyzed using one-way ANOVA, followed by a post hoc Tukey test. The data were tested for skewness kurtosis and normality with a Shapiro–Wilk test. Flow cytometry results were analyzed using a chi-squared test to determine a significant difference between the control and treated samples (G0/G1, S, G2/M phase percentages). A *p*-value < 0.05 was considered statistically significant for all methods.

## Data Availability

Data are contained within the article.
